# A randomised controlled trial of the use of aromatherapy and hand massage to reduce disruptive behaviour in people with dementia

**DOI:** 10.1186/1472-6882-13-165

**Published:** 2013-07-10

**Authors:** Chieh-Yu Fu, Wendy Moyle, Marie Cooke

**Affiliations:** 1 Centre for Health Practice Innovation, Brisbane, QLD 4111, Australia; 2 Griffith Health Institute, Brisbane, QLD 4111, Australia; 3 School of Nursing and Midwifery, Griffith University, 170 Kessels Road, Brisbane, QLD 4111, Australia

**Keywords:** Aggression, Agitation, Aromatherapy, Behaviour, Dementia, Massage

## Abstract

**Background:**

Aromatherapy and hand massage therapies have been reported to have some benefit for people with dementia who display behavioural symptoms; however there are a number of limitations of reported studies. The aim is to investigate the effect of aromatherapy (3% lavender oil spray) with and without hand massage on disruptive behaviour in people with dementia living in long-term care.

**Methods:**

In a single blinded randomised controlled trial 67 people with a diagnosis of dementia and a history of disruptive behaviour, from three long-term care facilities were recruited and randomised using a random number table into three groups: (1) Combination (aromatherapy and hand massage) (n = 22), (2) Aromatherapy (n = 23), (3) Placebo control (water spray) (n = 22). The intervention was given twice daily for six weeks. Data on residents’ behaviour (CMAI) and cognition (MMSE) were collected before, during and after the intervention.

**Results:**

Despite a downward trend in behaviours displayed not one of the interventions significantly reduced disruptive behaviour.

**Conclusions:**

Further large-scale placebo controlled studies are required where antipsychotic medication is controlled and a comparison of the methods of application of aromatherapy are investigated.

**Trial registration:**

ACTRN12612000917831

## Background

Physical restraints and antipsychotics are frequently used to manage disruptive behaviours displayed by people with dementia living in long-term care [1,2]. Both forms of restraint can increase the risk of falls, extrapyramidal symptoms, cerebrovascular adverse events, and metabolic syndrome [3]. Several complementary and alternative medicine (CAM) modalities have received attention as being potentially useful in the management of disruptive behaviours in people with dementia. Aromatherapy and hand massage offer an alternative approach to the risk of pharmacological intervention such as antipsychotics.

Aromatherapy can be absorbed through application to the skin or through the respiratory system. There is an assumption that the aroma and the constituents in the essential oil enter the bloodstream and combine to result in a psychological and physiological response [4]. Aromatherapy used to reduce agitated behaviours in people with dementia has predominately focused on essential oils believed to have a calming and sedative effect. Such studies have commonly used either Melissa officinalis (Lemon balm) or Lavender essential oil. It is believed that Lavender oil exerts a direct action on tryptophan, and helps the relaxation response [5].

A Cochrane systematic review of aromatherapy and dementia [6] identified only one study [7] that revealed a statistically significant effect of aromatherapy on behavioural and psychological symptoms of dementia (BPSD). In this study [7] 72 people with severe dementia, from eight nursing homes were randomly assigned to an intervention group (10% Melissa officinalis, or a placebo of sunflower oil. Both essential oils were combined with lotion and applied to the skin twice a day for a four-week period of treatment. Aromatherapy in this study was associated with a significant reduction in aggressive behaviours, as assessed by staff using the Cohen-Mansfield Agitation Inventory [8], Neuropsychiatric Inventory [9] and Dementia Care Mapping [10], in 35% of participants treated with melissa oil and in 11% of those receiving the placebo. The Cochrane review [6] identified methodological confounding effects that may have influenced the study results such as the participants were taking a range of pharmaceutical treatments that may have been altered during the trial and therefore influenced BPSD scores. They recommended further large-scale studies were required as one small trial was insufficient evidence for the efficacy of aromatherapy on BPSD.

Two further systematic reviews have also reviewed the use of aromatherapy. One of these (4) focused on aromatherapy use in the treatment of BPSD and the other focused more generally on aromatherapy for health care and as a result included BPSD [11]. Both systematic reviews suggest there are limited studies that demonstrate the effectiveness of aromatherapy and the majority of studies to date have been challenged by severe methodological limitations. Fung and colleagues [4] however, identified four studies, including the Ballard study (already described) that indicate aromatherapy might be regarded as a potentially effective treatment for BPSD (4). The three studies are outlined below.

A significant reduction in agitated behaviour using lavender oil was identified in a cross-over randomised trial in Hong Kong [12]. Seventy Chinese older people with moderate to severe dementia were recruited for a study of the effects of six-weeks of lavender or sunflower inhalations, with a two-week washout. Total mean scores of the Chinese version of the Cohen-Mansfield Agitation Inventory (CCMAI) showed a positive reduction from 24.68 to 17.77 (*p* < .001); mean scores on the Chinese version of the Neuropsychiatric Inventory (CNPI) also positively reduced from 63.17 (*SD* = 17.81) to 58.77 (*SD* = 16.74). The main limitation to this study was that raters were not blinded to the treatment offered.

Lavender oil also had a positive effect on BPSD in another study. A placebo controlled trial with 15 participants with severe dementia using 2% lavender oil aromatherapy steam every second day for a total of 10 sessions resulted in 60% (n = 9) of participants showing improvement, 33% (n = 5) showed no change and 7% (n = 1) showed a worsening of agitated behaviour when compared to the control group [13]. This study was limited by the small sample size.

The largest aromatherapy study to date was a double-blind parallel-group placebeo-controlled randomised trial across 3 centres in the UK using melissa oil [14]. In this study 114 participants were allocated to 1 of 3 groups: placebo medication and active aromatherapy; active medication and placebo aromatherapy or placebo of both. The Pittsburgh Agitation Scale [15] and Neuropsychiatric Agitation Inventory [9] were completed at 4 weeks and 12 weeks follow-up. There was no evidence that melissa oil aromatherapy was superior to placebo or donepezil, an anticholinesterase, in reducing BPSD. There was a change from baseline to week 12 in quality of life (QOL), with the aromatherapy group experiencing the best outcome.

A number of small studies have also combined essential oils in the belief that this would improve outcomes. However, only one study has reported a significant reduction in agitated behaviours using a combination of essential oils including lavender, camomile, rosemary and marjoram [16]. Essential oils were provided in a footbath, massage on upper body and hands or on pillows. However, the findings were based only on observations. This study was further limited by the lack of blinding, small sample size and data collection undertaken by care staff [16]. In addition the application of aromatherapy by massage or touch, rather than inhalations may also confuse the findings as there is a small amount of evidence suggesting that massage and touch by themselves may influence BPSD [17,18].

In summary although the findings of the studies are limited they suggest that aromatherapy and massage may help to reduce BPSD in people with dementia. This current study, aimed to overcome the following limitations of previous studies; a clear explanation of the protocol including length of treatment and blinding of the raters, use of trained raters and an adequately powered sample to investigate the effect of aromatherapy, the aromatherapy oil sprayed rather than massaged to reduce the potential impact of massage, and an intervention group using aromatherapy combined with hand massage to identify the impact of the combined intervention on BPSD in people with dementia in long-term care (LTC) facilities. We conducted a single blinded randomised controlled trial to assess the effect of aromatherapy (3% lavender oil spray) with and without hand massage on disruptive behaviour in people with dementia living in long-term care. Lavender oil was chosen because it was shown to have the most consistent effect on aggressive and nonaggressive behaviours [12,13].

## Method

### Study design

A total of 67 older people were randomised to receive six weeks of twice daily aromatherapy, or aromatherapy and hand massage, or water spray (placebo control). Primary outcomes assessed before, during, and post intervention were aggression and agitation. All recruitment and intervention protocols took place between February and December 2006. Ethical approval to conduct the study was obtained from the University Human Research Ethics Committee and the management of each aged care facility.

### Study population

Participants were recruited from three LTC facilities located in Brisbane, Australia, owned and operated by the one provider and with a combined total of 284 beds. The facility environments, staffing models and philosophies of care were similar across the three facilities. To ensure the intervention was targeted to residents who it was assumed would get the most benefit from the intervention participants had to meet the following inclusion criteria: 1. aged 60 or over to avoid recruitment of persons with early onset dementia; 2. living in a participating nursing home for at least three months to avoid potential effects from transition to the nursing home; 3. cognitive functional impairment indicative of a dementia condition; MMSE score of 24 out of 30 or less; and features of Alzheimer’s disease according to American Psychiatric Association DSM-IV-TR [19]; 4. a documented history of a minimum of two weeks of agitation or aggression in total (consecutively or 14 single days), within the past three months; 5. a documented history of physical and/or chemical restraint for agitation and aggression, including PRN (as required) medication; 6. consent for participation from resident’s family or health-attorney; 7. no known allergic reaction to lavender oil; and 8. no recent skin tears, lacerations, bruises, or redness and swelling that might interfere with hand massage. Exclusion criteria were: 1. a diagnosis of schizophrenia or mental retardation to avoid the complication of dual diagnoses impacting on treatment effect; 2. expected to be transferred to another residential facility within the next 3 months.

We invited the facility care managers to identify residents who appeared to meet the selection criteria from a population of 284. They then sent information and informed consent forms to next of kin of the 165 residents identified. Randomisation assignments were given to participants following baseline testing; these were generated using a random number table and a person not involved in the study randomised participants into three groups in each residential care facility. Participant demographics were obtained from the facility manager who copied information required for the study from resident records.

### Interventions

Aromatherapy spray was used because it is a simple and convenient way to provide the aromatherapy treatment and control treatment dose. It also allowed the potential confounding effect of massage to be controlled for in this current study, with aromatherapy intervention groups with and without massage. In comparison, a vaporizer would be difficult to quarantine and would result in staff recognizing treatment groups. Given the likelihood that participants might have compromised olfactory systems [20], it was decided that a direct spray onto individuals’ upper chest would be an effective delivery method.

This is the first recorded study to apply an essential oil spray to people with dementia, and therefore there was no precedent in the literature to follow when determining the essential oil spray dosage or an effective treatment period. Literature was consulted to help determine a suitable dosage regimen and frequency of application [7,21-23]. Aromatherapy guidelines were followed to reduce the risk of skin irritation, the guidelines recommend a maximum concentration of 3% essential oil as a safe dosage for skin use [23,24]. A 3% lavender mist, consisting of 75 drops of pure 100% lavender oil was mixed with 4 ml essential oil solubiliser and 125 cc purified water. All bottles of oil were stored out of direct sunlight.

An allergic skin test was carried out before commencing the intervention and was applied to participants’ inner arm. The aromatherapy intervention consisted of three sprays of lavender mist applied to the participants’ chest within a 30cm distance, avoiding the face and eyes. The control group received water mist sprayed in the same way. The length of time for the hand massage followed the protocol of Snyder *et al*. [22] which found a postive effect of agitation on people with dementia who had received five minutes of hand massage twice a day for 10 days: each hand was massaged for 2.5 minutes.

The interventions were administered by one researcher and six trained research assistants. All treatments were given twice a day, at two time periods, 9 am to 11 am and 2 pm to 4 pm, seven days a week for six weeks. Participants received treatments in a quiet and private environment, such as the participant’s room in an attempt to keep staff and family blind to the intervention type. If necessary, curtains and folding screens were used to screen participants from the view of the nursing staff.

### Outcome measures

All primary outcome measures were assessed by facility staff blind to treatment assignment. A cognitive assessment was undertaken at baseline and at the end of the six week intervention, using the Mini Mental State Examination (MMSE) [25]. The MMSE is the most widely used instrument to measure cognitive functioning and has been shown to have an excellent test/retest reliability of .89, and internal consistency of .83 [26]. The maximum score is 30 points. An MMSE score from 19 to 24 points indicates mild cognitive function impairment, a score from 10 to 18 indicates moderate cognitive impairment; and a score less than 10 indicates severe cognitive impairment [27].

Participants’ aggressive/non aggressive and agitated behaviours were measured using the Cohen-Mansfield Agitation Inventory-Short Form [8]. The CMAI-SF was administered five times in the study: 1. within the month prior to the intervention; 2. at the end of the second week of intervention; 3. at the end of the fourth week of intervention; 4. at the completion of the intervention, at the end of the sixth week; and 5. six weeks after the completion of the intervention, in week 12.

The Cohen-Mansfield Agitation Inventory (CMAI-SF) is an internationally validated instrument that measures behavioural disturbance in people with dementia in the previous two weeks. The items are scored on a five point frequency scale, from 1 “Never”, to 5 “A few times an hour or continuing for half an hour or more”. The CMAI-SF is completed by caregivers and takes approximately 10 minutes to complete. In the current study, to accommodate staff leave, a trained research assistant (rater) asked small groups of staff who were regular carers of the individual to rate the individual’s behaviour over the previous fortnight. The group discussed the rating to ensure there was consensus in the rating. At anyone rating session there were always a minimum of two carers who had previously rated the individual. Retrospective reviews of each participant’s functional needs were assessed from patient record data. This data recorded assessment data, including type and amount of assistance required for daily living.

### Statistical analysis and sample size

The sample size calculation for this study was based on a previous study [7] that explored the effectiveness of aromatherapy on agitated behaviour in older adults. This study was chosen as the authors also used the CMAI as the primary outcome measurement and at the time there was no other comparative study that used lavender oil. The Ballard study found that the change in CMAI scores post-intervention were 23.1 (SD = 12.85) for the intervention group, and 7.3 (SD = 17.1) for the control group, with an effect size of f = 0.52737. Using this reported effect size, G*Power a priori calculations, a sample size of 45 (15 in each group) to obtain a power of 0.95, with an α of .05, using a repeated measures ANOVA analysis framework was needed. Primary outcomes were percentage change from baseline to 6 weeks. All primary analyses were conducted according to the intention to treat (ITT) approach, which is to include the data of all randomised participants regardless of whether they finish the treatment [28]. Participants’ missing single items of baseline data were replaced by stochastic regression imputation (SRI). SRI substitutes missing data with mean and random value.

Responses were coded and entered into the Statistical Package for Social Sciences (SPSS) program, version 15.0. Data were checked for completeness and inconsistencies to avoid entry errors [29]. Given the abnormal distribution of test scores and the ordinal properties of the scale items and scores, a nonparametric Kruskal-Wallis H test (the nonparametric equivalent of ANOVA) was used to identify statistically significant outcomes [29]. Descriptive statistics were used to analyse the demographic data.

## Results

**Figure 1:**
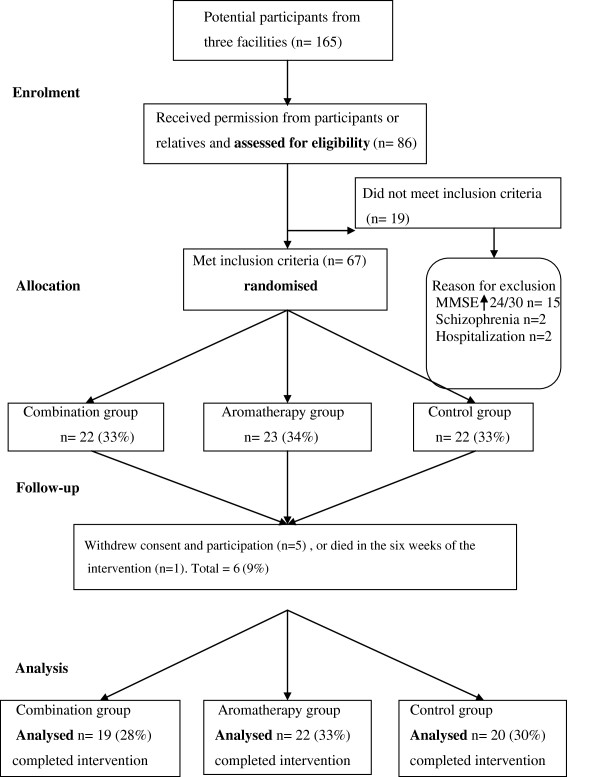
Flow chart of participants’ progress through randomised trial.

A total of 165 potential participants were identified from the three facilities. Permission to assess for eligibility was received from 86. Of these 19 did not meet the inclusion criteria. As a result 67 residents who met the selection criteria were recruited from the three facilities: 29 from Facility 1; 16 from Facility 2; and 22 from Facility 3. The participants’ age, frailty, and diagnoses all created participation challenges. One male resident died in the first week of the study, and as no data were collected he was excluded. Five participants or their relatives withdrew consent for participation and data during the six weeks of the intervention stage of the study. Withdrawal of consent was related to family wanting reassurance their family member was in the intervention rather than control group and the team being unable to reassure the family. The data for these individuals was also excluded [28]. A visual flowchart of participants’ progress through the randomised trial is presented in Figure 1.

Table 1 summarises participant demographics. Participants ranged in age from 61 to 93, with a mean age of 84 (*SD* = 6.36). The majority ranged in age from 80 to 89 (n = 43, 70.5%), regardless of which facility they lived in. Close to 50% (n = 28) lived in a high care nursing home setting: 31% (n = 19) lived in special care units. Nearly 90% (n = 53) relied on nursing staff to assist them with daily activities, such as feeding and hygiene. Most participants had at least one or two sensory deficits: some vision impairment was a health problem for a large majority (n = 51, 83.6%); followed by hearing impairment (n = 25, 41%); and chronic pain (n = 19, 31.1%). More than one-third (n = 25, 41%) walked with an assistive appliance, and 18 (29.5%) participants were chair-fast (not mobile). Fifteen (51.7%) of those who were chair-fast because of a physical condition also had a severe cognitive impairment. An Optimal Scaling (SPSS) representation of the demographic profile of the participants of all three facilities did not reflect differences between the clientele of the facilities.

**Table 1:** Participant demographics

	**n (%)**
**Gender**	
Male	25 (41%)
Female	26 (59%)
**Age**	
60-69	1 (1.6%)
70-79	10 (16.4%)
80-89	43 (70.5%)
90 & older	7 (11.5%)
**Living setting**	
Hostel	14 (23%)
Nursing home	28 (46%)
Special care unit	19 (31%)
**Care level**	
High care	53 (86.9%)
Low care	8 (13.1%)
**Sensory deficits**	
(1 or more could be chosen)	
Hearing	25 (41.0%)
Vision	51 (83.6%)
Pain	19 (31.1%)
**Mobility**	
Mobile	18 (29.5%)
Wheelchair	3 (4.9%)
Walking stick	2 (3.3%)
Mobile Walker	20 (32.8%)
Chair-fast (not mobile)	18 (29.5)

Table 2 sets out the participants’ diagnoses and their cognitive impairment. Almost half (n = 29) had been diagnosed with ‘dementia’, the next largest group were those diagnosed with ‘Alzheimer’s disease’ (AD) (n = 16, 26.2%). Eight participants did not have a definitive diagnosis. They did, however, have a cognitive impairment and met the DSM-IV-TR criteria for AD [19]. The largest proportion (n = 29, 47.5%) of participants had MMSE scores which placed them within the category of severe cognitive impairment. Fourteen (23%) participants with severe cognitive impairment scored 0 on the MMSE, indicating they had not been able to respond to any of the MMSE questions. Of these 14 participants, 6 (42.9%) were in the combination group (aromatherapy and hand massage), 3 (21.4%) were in the aromatherapy group, and 5 (35.7%) were in the control group.

**Table 2:** Participants’ diagnoses and cognitive impairment

	**n (%)**
**Diagnosis**	
Cognitive impairment	8 (13.1%)
Dementia	29 (47.5%)
Alzheimer’s disease	16 (26.2%)
Vascular dementia	3 (4.9%)
Multi-infarct dementia	2 (3.3%)
Other (e.g. Toxic dementia)	3 (4.9%)
**Baseline-Mini-mental status (score)**	
Mild (19–24)	9 (14.8%)
Moderate (10–18)	23 (37.7%)
Severe (0–9)	29 (47.5%)

Table 3 shows at baseline the majority of participants displayed verbal agitation, such as constant requests for attention or help (n = 43, 70.5%), and physical agitation (n = 53, 86.9%), such as wandering. A significant proportion of the participants displayed verbal aggression (n = 25, 41%), and 18 (29.5%) displayed physical aggression, such as hitting, kicking, throwing objects and tearing up materials. More than half (n = 31, 50.8%) were regularly given an analgesic such as paracetamol or aspirin. A major proportion (n = 50, 82%) of participants were subject to both physical and chemical restraint. Over 54% (n = 11) of the 19 participants who lived in special care units (SCU) were on antipsychotic medication.

**Table 3:** Participants’ aggressive behaviours, restraints, and regular antipsychotic medications

	**n (%)**
**Aggressive behaviours**	
(can chose more than 1)	
Verbal agitation	43 (70.5%)
Physical agitation	53 (86.9%)
Verbal aggression	25 (41.0%)
Physical aggression	18 (29.5%)
**Restraints**	
No restraint used	1 (1.6%)
Physical restraint	1 (1.6%)
Chemical restraint	9 (14.8%)
Both physical and chemical	50 (82.0%)
**Regular antipsychotic medicine**	
(1 or more could be chosen)	
Risperidone	9 (14.8%)
Haloperidol	5 ( 8.2%)
Other (e.g. Temazepam, Zolpidem)	14 (22.8%)

The CMAI-SF showed a high reliability for the five times it was used with a Cronbach’s alpha estimate from .87 to .91 and the Guttman split-high reliability estimates from .78 to .89. Participants’ aggressive behaviours were measured at five points in time, using the CMAI-SF (see Table 4). In all subscales, apart from ‘*Inappropriate dress or disrobing’* the mean scores across the five time points reduced, although not significantly. The biggest decrease in mean score occurred in the nonaggressive subscale of ‘*General restlessness, performing repetitious mannerisms, tapping, strange movements*’ where the baseline mean score decreased from 3.07 (SD = 1.47) to 2.28 (SD = 1.53). The average mean scores of the data recorded at five time points for the total CMAI–SF item score was 1.96 (*SD* = .72) and the four CMAI-SF sub scales were: physical nonaggressive 2.11 (*SD* = .95); aggressive 1.77 (*SD* = .68); verbal agitation 2.33 (*SD* = 1.09); and hiding and hoarding things 1.75 (*SD* = .96).

**Table 4:** Data summary for five time points of the Cohen-Mansfield Agitation

**CMAI subscales/ five time points**	**Baseline**	**Week 2**	**Week 4**	**Week 6**	**Post-test**
***(n = 61)***	**M (SD)**	**M (SD)**	**M (SD)**	**M (SD)**	**M (SD)**
**Nonaggression**					
05. Pace, aimless wandering, trying to get to a different place	2.48 (1.66)	2.30 (1.50)	2.38 (1.54)	2.33 (1.51)	2.18 (1.58)
06. General restlessness, performing repetitious mannerisms, tapping, strange movements	3.07 (1.47)	2.61 (1.48)	2.48 (1.50)	2.41 (1.45)	2.28 (1.53)
07. Inappropriate dress or disrobing	1.74 (1.05)	1.79 (1.14)	1.67 (0.96)	1.77 (1.06)	1.77 (1.16)
08. Handling things inappropriately	2.0 (1.33)	1.80 (1.03)	1.79 (1.14)	1.75 (1.22)	1.56 (0.98)
**Aggression**					
01. Cursing or verbal aggression	2.61 (1.28)	2.36 (1.17)	2.20 (1.21)	2.13 (1.23)	2.20 (1.15)
02. Hitting, kicking, pushing, biting, scratching, aggressive spitting	1.84 (1.07)	1.77 (1.01)	1.57 (0.97)	1.62 (0.90)	1.72 (1.02)
03. Grabbing onto people, throwing things, tearing things or destroying property	2.02 (1.23)	1.90 (1.12)	1.98 (1.27)	1.82 (1.04)	1.89 (1.07)
04. Other aggressive behaviours or self- abuse	1.69 (1.04)	1.59 (1.02)	1.59 (1.02)	1.54 (1.03)	1.43 (0.87)
14. Screaming	1.36 (0.93)	1.21 (0.76)	1.31 (0.74)	1.30 (0.72)	1.28 (0.64)
**Agitation**					
09. Constant request for attention or help	2.38 (1.45)	2.20 (1.45)	2.21 (1.52)	1.98 (1.32)	2.0 (1.39)
10. Repetitive sentences, calls, questions or words	2.82 (1.53)	2.56 (1.46)	2.49 (1.50)	2.34 (1.34)	2.41 (1.42)
11. Complaining, negativism, refusal to follow directions	2.75 (1.36)	2.38 (1.23)	2.25 (1.35)	2.03 (1.14)	2.13 (1.38)
**Hiding & hold things**					
13. Hiding things, hoarding things	2.08 (1.44)	1.74 (1.06)	1.59 (1.01)	1.67 (1.04)	1.69 (1.15)

Note: The scale ranged from 1 (Never) to 5 (A few times an hour or continuous for half an hour or more). Question 12 Strange noise, did not fit into any sub-groups; therefore it was excluded from the study.

A Kruskall-Wallis H test showed that both the aromatherapy and placebo treatment groups had reduced aggressive behaviours at the end of week six of the intervention (p < .05). There were no significant differences in participants’ mean scores for the CMAI across all periods (p < .05).

### Adverse events

No adverse events were reported in the trial.

## Discussion

The results of this study did not demonstrate that the two CAM modalities, aromatherapy alone, and aromatherapy combined with hand massage had any statistically significant effect on reducing participants’ aggression and/or agitation. This is contrary to the findings of the two earlier lavender oil studies by Holmes *et al*. [13] and Lin *et al*. [12] that found a reduction in agitation according to the CMAI [8] and the Pittsburgh Agitation Scale [9]. In the current study the CMAI ‘agitation’ sub-scale had the highest scores. All sub-scales showed a slight reduction in aggressive behaviours at the second, third and fourth data collection point but none of these reductions in aggressive behaviours was statistically significant. There is no clear explanation why the results are different in this study but it is suggested that this could be related to the application of the aromatherapy oil. In this study the aromatherapy oil was sprayed onto participants’ upper chest, whereas in previous studies the aromatherapy was massaged in or given as an inhalation. Further research is needed to compare the different type of application of essential oils. In addition this study did not control for antipsychotic use and their use could have had a cofounding effect on CMAI scores. Furthermore, although this study did not demonstrate significant results there were individuals who obviously benefited from the intervention. This study supports the need to tailor interventions to the needs and wishes of individuals. Further research is required to identify if aromatherapy and/or hand massage is more effective in individuals who had prior enjoyable experiences of aromatherapy and/or massage.

The study has methodological strengths that aimed to avoid the limitations of previous studies in the area. The research design, three treatment groups, including a control group avoided a short period of washout and the lack of a comparative [30,31]. In addition, the six-week intervention provided an appropriate length of treatment for determining a dose–response [32,33]. Using a clearly identified concentration of a single essential oil (3% lavender angustifolia) addressed limitations in previous studies, which often failed to report standardised dosage information [33]. The collection of pre-intervention participant data provided baseline information and allowed for comparison with each post-test data set.

### Study limitations

This study has several limitations. First, the sample size somewhat limits its generalisability. Secondly, participants’ level of olfactory functioning may have affected aromatherapy effectiveness. There is evidence that people with dementia often have olfactory dysfunction. In particular, those people with dementia with Lewy bodies [20,34]. An olfactory function test was not undertaken because of participants’ various levels of cognitive impairment and disruptive behaviour. Despite providing training for staff in each facility in the use of the CMAI, staff continued to make subjective judgments when asked to record participants’ behaviours. Nursing staff often stated that they viewed aggressive behaviours as a routine aspect of caring for people with dementia, something they had to deal with every day. Although this had the potential to impact on the CMAI recordings the researchers aimed to limit reliability issues by the rater asking a small group of carers for their ratings of participant’s behaviour. This provided the opportunity for carers to discuss together and confirm their perceptions of individual behaviours. Furthermore aromatherapy treatment was used as an adjunct to current interventions, both pharmacological and non-pharmacological and no control was exercised over participants’ routine medications. Over 45% of the participants (n = 28) were on regular antipsychotic medication and this prescribed medication might have affected their behaviours [12,19]. Although the intervention was performed in the private areas of the care facility staff or family may have become aware of the nature of the treatment given to participants if the lavender oil odor remained in the environment following the treatment. However, given that staff and family on occasion referred incorrectly to the treatment a participant was receiving this seemed unlikely.

## Conclusions

This study did not identify significant improvements in aggressive behaviours of people with dementia who received aromatherapy or who received aromatherapy in combination with hand massage. These findings are important for practitioners to consider when deciding upon whether to introduce aromatherapy. Practitioners must consider individual needs, including assessment of the triggers behind behaviours of concern and evaluate the effect of any interventions, including aromatherapy or massage put into place to manage the behaviour. The findings are also important for researchers to consider in their design of any future studies and indicate that further large sample studies are required.

## Competing interests

The authors declare that they have no competing interests.

## Authors’ contributions

CF conceived of the study and its design, coordinated the research and trained RAs. WM and MC oversaw the study and assisted in providing methodological advice. All authors contributed to the drafting of the manuscript. All authors read and approved the final manuscript. No writing assistance was utilized.

## Pre-publication history

The pre-publication history for this paper can be accessed here:

http://www.biomedcentral.com/1472-6882/13/165/prepub
